# Editorial: Chromatin Stability and Dynamics: Targeting and Recruitment of Chromatin Modifiers

**DOI:** 10.3389/fpls.2021.678702

**Published:** 2021-05-31

**Authors:** Sara Farrona, Iva Mozgová, Rafal Archacki, Juan Armando Casas-Mollano

**Affiliations:** ^1^Plant and Agricultural Biosciences Centre-Ryan Institute, National University of Ireland Galway, Galway, Ireland; ^2^Biology Centre, Czech Academy of Sciences, Ceske Budejovice, Czech Republic; ^3^Laboratory of Systems Biology, Faculty of Biology, University of Warsaw, Warsaw, Poland; ^4^Institute of Biochemistry and Biophysics PAS, Warsaw, Poland; ^5^BioTechnology Institute, University of Minnesota Twin Cities, St. Paul, United States

**Keywords:** chromatin dynamics, histone modifications, chromatin remodeling complexes, transcriptional regulation, plant development and responses

Chromatin organizes nuclear genome in the restricted space of the nucleus and contributes to all nuclear processes which occur in the absence of internal membranes. Chromatin structure is highly dynamic allowing the unconstrained but controlled reprogramming of nuclear processes, including gene expression, in response to internal and external cues. This is particularly important in plants that, as sessile organisms, constantly need to modify their development and growth (Santos et al., 2020).

The articles published through this Research Topic present new data or discuss current knowledge related to our understanding of chromatin dynamics and its relevance for the regulation of plant growth and environmental responses. Furthermore, novel techniques to deepen our understanding and visualization of chromatin dynamics are also presented.

## Histone Modifications

As key structural components of the chromatin, histones are the main target of regulatory complexes and are subjected to an array of posttranslational modifications. One of the best studied histone modifications is methylation, from which histone H3 lysine 9 methylation has been shown to be associated to the silencing of genomic parasites and repetitive sequences in plants and most eukaryotes (Xu and Jiang). Recent progress in decoding the functions of histone H3 lysine 9 di-methylation (H3K9me2) in the model plant *Arabidopsis thaliana* (Arabidopsis) are discussed by Xu and Jiang. In their review, Xu and Jiang, give an overview of the methyltransferases involved in the methylation of H3K9 and how this modification is properly deposited at its target genomic regions. Current knowledge on the readers and functional outcomes of H3K9 methylation are also highlighted (Xu and Jiang). In an original research article, Demidov et al. shed light on the functions of the phosphorylation of the centromere-specific histone 3 (CENH3), a variant that replaces the canonical histone H3 in centromeric regions. Using a modification-specific antibody, Demidov et al. showed that Arabidopsis CENH3 is phosphorylated at serine 65 (CENH3 pS65) *in vivo*. CENH3 pS65 may have a role in reproductive development as suggested by its enrichment in floral buds and the defects in reproductive tissues, and plant growth and development, caused by perturbations in this modification. The authors also provide evidence that the kinase Aurora3 may be involved in the phosphorylation of CENH3 S65 (Demidov et al.).

## Technical Advances

*In vivo* visualization of specific loci within chromatin is pivotal for understanding chromatin dynamics in plant nucleus in response to external or internal cues. Addressing the question of whether rapid ion-based signaling and changes in membrane potential can result in changes in chromatin dynamics, Matzke et al. have developed tools that allow *in-vivo* monitoring of concomitant changes in pH and chromatin dynamics at individual genomic loci in Arabidopsis. To monitor changes in pH elicited upon root treatment by extracellular ATP (eATP), the system employs the pH sensor protein SEpHluorinA227D targeted to different cellular membranes (including plasma membrane or inner nuclear membrane) or to specific chromatin loci tagged by Tet or Lac operator (Tet/Lac-O) sequences. This is combined with Tet/Lac-O-targeted fluorescent proteins that allow monitoring chromatin dynamics at these loci. Using the system, the authors show that addition of eATP can lead to reduction of pH at sites of chromatin-bound proteins, which correlates with changes in dynamics of chromatin-bound proteins (Matzke et al.). In a different article of this Research Topic, the groups of Holger Puchta and Andreas Houben use MS2 or PP7 aptamers, short RNA oligos that can be recognized by RNA-binding proteins fused to a reporter, that are incorporated into sgRNA to amplify the GFP signal for *in vivo* labeling of plant telomeric sequences using the CRISPR/deactivated Cas9 (dCas9) system. The system improved the detection possibilities and signal/noise ratio of telomeres in transiently transformed *Nicotiana benthamiana* (Khosravi et al.). Unfortunately, it proved not functional in stably transformed plants, including *N. benthamiana*, Arabidopsis or *Daucus carota* (carrot), perhaps, as the authors speculate, due to the interference of stable CRISPR/dCas9 RNP binding to telomeres with plant growth and development (Khosravi et al.). Nevertheless, the use of aptamers within sgRNA is a promising strategy for signal amplification and sensitivity during *in vivo* imaging of plant chromatin.

## Chromatin-Related Complexes

Protein-protein interactions of nuclear components to form different type of multimeric complexes play a key role in the regulation of chromatin dynamics. Grasser has reviewed our current knowledge of one of these complexes, the heterodimeric histone chaperone FACT that is well-conserved among eukaryotic organisms and controls nucleosome assembly/disassembly linked to some of the most important DNA-related processes (Formosa and Winston, 2020). In this review article, the role of Arabidopsis FACT in the regulation of transcription, particularly during elongation, is discussed (Grasser). In addition, the impact of FACT on plant developmental switches through the regulation of the expression of key developmental genes, such as *FLOWERING LOCUS C* (*FLC*), master repressor of flowering, and *DELAY OF GERMINATION 1* (*DOG1*), key repressor of germination, is highlighted. However, how this histone chaperone complex is recruited to the chromatin or what poses specific genes to be more dependent on FACT-mediated regulation are still open questions that will require further investigation (Grasser). The laboratories of José A. Jarrillo and Manuel Piñeiro have contributed to our understanding of the activities of the NuA4 complex in plant chromatin remodeling. In this Research Topic they provide an overview of the Arabidopsis putative NuA4 complex during flowering and also describe the essential role of this complex in other cellular processes (e.g., stress and hormone responses) (Espinosa-Cores et al.). Furthermore, the complex scenario of the interactions of NuA4 with accessory proteins to form different complex variants in other organisms is profusely covered and used to make an elegant comparison of the situation in Arabidopsis (Espinosa-Cores et al.).

## Metabolism and Chromatin

Chromatin-based mechanisms are involved in transcriptional regulation of virtually all major developmental and growth processes, including key metabolic pathways. The research article by Meng et al. reports that in maize, ZmCHB101 is necessary for proper physiological responses and gene expression changes upon nitrate treatment. ZmCHB101 is a close homolog to Arabidopsis SWI3D, one of the subunits of the SWI/SNF remodeling complex (Meng et al.). The authors identified two *ZmCHB101* target genes involved in nitrate transport, and observed that nucleosome occupancy and selected histone modifications at these loci were affected in ZmCHB101 RNAi lines. Interestingly, the presence of nitrate seems to negatively affect ZmCHB101 binding to these targets, which in turn may facilitate the binding of another nitrate-associated regulator, ZmNLP3.1 (Meng et al.). How the occurrence of nitrate in the cell is exactly translated into observed downstream effects is an intriguing question that needs further investigation. The connection of metabolism with epigenetics is reviewed in this Research Topic by Leung and Gaudin. The review discusses current knowledge about how metabolites modulate chromatin-modifying machineries. Specially, it focuses on acetylation and methylation and the key substrates for these modifications, acetyl-coenzyme A and S-adenosylmethionine, which provide most of the current experimental data. However, as the authors emphasize, DNA and histones (but also non-histone proteins, RNAs and various metabolites) have been shown to be subjected to a myriad of different chemical modifications (Leung and Gaudin). Collectively, they may form a crucial link between the metabolic status of the cell, largely dependent on environmental conditions, and the epigenetic information with its output on gene expression. Plants, with their lifestyle and ability to produce vast amounts of secondary metabolites are especially interesting organisms to study these phenomena.

## Conclusion

The diverse scope of themes covered by the articles in this Research Topic finely reflects the dynamic nature of the plant chromatin research field. Some of the most recent advances in this area presented during the 2019 European Workshop on Plant Chromatin (EWPC) have been collected and summarized in a review article in this Research Topic (Moreno-Romero et al.). We envisage that these and future advances in the chromatin and epigenetic field will become essential for understanding the fundaments of chromatin dynamics and, very importantly, to bridge the gap between this knowledge and its implementation for the epigenetic control of plant traits.

## Author Contributions

All authors listed have made a substantial, direct and intellectual contribution to the work, and approved it for publication.

## Conflict of Interest

The authors declare that the research was conducted in the absence of any commercial or financial relationships that could be construed as a potential conflict of interest.
